# Caecal Perforation from Primary Intestinal Tuberculosis in Pregnancy

**DOI:** 10.1155/2017/2173724

**Published:** 2017-08-17

**Authors:** Soe Lwin, Nina Lau Lee Jing, Haris Suharjono, Mardiana binti Kipli, Tin Moe Nwe, Myat San Yi, Lucas Luk Tien Wee

**Affiliations:** ^1^Department of Obstetrics & Gynecology, Faculty of Medicine and Health Sciences, UNIMAS, Kota Samarahan, Malaysia; ^2^Department of Obstetrics & Gynecology, Sarawak General Hospital, Kuching, Malaysia; ^3^Department of Basic Health Sciences, Faculty of Medicine and Health Sciences, UNIMAS, Kota Samarahan, Malaysia

## Abstract

The incidence of tuberculosis (TB) is rising worldwide, despite the efficacy of the BCG vaccination. Populations at greatest risk of contracting TB are migrant communities, as well as immunocompromised individuals. The diagnosis of extrapulmonary TB (EPTB) can often present as a diagnostic conundrum, due to its nonspecific and varied presentation, often mimicking inflammatory bowel disease or malignancy. We present a case of caecal TB in pregnancy, which resulted in caecal perforation, a right hemicolectomy, and severe preterm delivery. The aim of this case report is to discuss the diagnosis of extrapulmonary TB, as well as its subsequent management in pregnancy.

## 1. Introduction

TB remains a global epidemic, with an estimated 10.4 million new cases diagnosed worldwide in 2015. Of these, there was a slight male preponderance, 56% of all new cases, as compared to 34% for women. TB in children contributed to the remaining 10% of new cases. TB mortality rates are alarmingly high and in 2015, approximately 1.8 million deaths were attributable to TB, amongst whom those coinfected with Human Immunodeficiency Virus (HIV) made up a quarter of total deaths [1].

The diagnosis of EPTB can often present as a diagnostic conundrum, due to its nonspecific and varied presentation, often mimicking inflammatory bowel disease or malignancy. Immunocompromised individuals have been found to be more susceptible to EPTB [2] and this may explain its relative predominance in the young female population, as pregnancy is a state of relative immunocompromise.

Abdominal TB accounts for 11% of all EPTBs, with the most common site of involvement being the ileocaecal region [2]. Postulations for the propensity for ileocaecal involvement have included increased physiological stasis and fluid absorption and reduction in digestive activity, as well as the abundance of lymphoid tissue in this region (Peyer's patches) [3].

Symptoms of abdominal TB include chronic abdominal pain, abdominal distension, fever, night sweats, and loss of appetite, as well as rapid and significant weight loss. In diagnosing abdominal TB, a high index of suspicion is required, as the clinical signs are often nonspecific. Pregnancy further confounds clear diagnosis, as pregnancy related abdominal pains such as preterm labour and placental abruption have to be considered. Medical and surgical causes of abdominal pain, such as inflammatory bowel disease, appendicitis, acute pyelonephritis, and colorectal cancer, have to also be excluded.

Diagnostic and treatment modalities, even in pregnancy, are similar to the nonpregnant population.

## 2. Case Presentation

Patient was 31-year-old female and 15 weeks into her second pregnancy, when she commenced her antenatal care. Her Body Mass Index (BMI) at booking was only 15.53 kg/m^2^, with a booking weight of 36 kg. She had been experiencing intermittent, dull, right-sided abdominal pain that was associated with diarrhea, for 3 months and in this span of time, her weight had decreased markedly by 20 kg. Her primary healthcare provider at the district maternal and child health clinic subsequently treated her for acute gastroenteritis twice in the following weeks, but she experienced no improvement in her symptoms.

At 29 weeks and 6 days of gestation, she was admitted to a district hospital, complaining of acute right-sided abdominal pain that radiated to the right lumbar region. She also complained of dyspnoea and near-syncopal episodes, as well as a reduced effort tolerance the preceding week. She had been compliant to her oral hematinics therapy and denied any history of dysuria, haematuria, haematemesis, or haematochezia. A recent peripheral blood film revealed normochromic, normocytic anaemia, and serum ferritin was normal.

On physical examination, she appeared pale and she was febrile. On palpation, her fundal height corresponded to 30 weeks of gestation. Tenderness and guarding were elicited at the right lumbar region. However, she did not exhibit any signs of preterm contraction and renal punch was negative. Transabdominal scan revealed a singleton fetus, with parameters corresponding to her gestation.

She had hemoglobin of 7.4 g/dl and a white cell count of 15.87 × 10^9^. Broad-spectrum antibiotic was commenced. She was also transfused with 1 pint of packed cells and she was referred to both the obstetrics and surgical teams of a tertiary hospital.

An urgent ultrasound abdomen was arranged, which revealed a heterogeneous collection in the subhepatic region and multiple enlarged mesenteric lymph nodes. A diagnosis of likely perforated appendicitis was made and she underwent an emergency laparotomy.

The intraoperative finding was that of a perforated caecal tumour. The tumour was firm and hard with irregular margins and surface. There was evidence of multiple enlarged mesenteric lymph nodes. A right hemicolectomy was performed. Appendix was normal. She received 3 pints of packed cells transfusion and 4 units of fresh frozen plasma. Postoperatively, she was transferred to the Intensive Care Unit (ICU), where she recovered well and was transferred to the general ward after 2 days. 7 days after surgery, she developed spontaneous preterm contractions and delivered a baby boy of 1.2 kg, at 31 weeks of gestation with Apgar score of 7 in one minutes and 9 in five minutes. The baby was given Bacillus Calmette-Guerin (BCG) vaccination immediately after delivery as routine procedure.

The histopathological examination showed caecal tuberculosis. She was referred to the Infectious Disease (ID) team and started on anti-TB medication. She denied any pulmonary TB contact. Her pulmonary TB work-up including chest X-ray, sputum acid fast bacilli (AFB), and Mantoux test was negative. Screening tests for HIV were negative.

She developed surgical wound breakdown at 14 days postoperatively and secondary suturing was performed. Her baby was initially admitted to the Neonatal Intensive Care Unit (NICU) for extreme prematurity but was subsequently discharged well with good catch-up growth and a negative Tuberculin skin text/Mantoux test. At a subsequent follow-up upon completion of 6 months of anti-TB medications, she had put back most of her weight and her baby was assessed to have achieved age-appropriate developmental milestones.

## 3. Discussion

EPTB is thought to be a result of reactivation of a prior dormant TB focus. Gastrointestinal TB is the sixth commonest site of extrapulmonary disease [4, 5]. Its occurrence is attributed to four mechanisms: hematological, swallowing of infected sputum in patient with active pulmonary TB, ingestion of contaminated milk or food, and contiguous transcoelomic spread from adjacent organs [6]. The ileocaecal region is affected in 75% of abdominal TB and this can present with perforation, abdominal mass, obstruction, and malabsorption [2, 7–9]. Viscus perforation due to TB is very rare and occurs more commonly in immunosuppressed patients [10]. Pregnancy is a physiological state of relative immunosuppression. Hence, the risk of viscus perforation after contracting abdominal TB in pregnancy may be increased, as was the case in our patient.

Diagnosis abdominal TB is challenging, presenting in a myriad of nonspecific ways, including abdominal pain, weight loss, loss of appetite, fever, diarrhea, constipation, or haematochezia. Diagnostic modalities that may help in the diagnosis of abdominal TB are outlined below.

### 3.1. Blood Investigations

The full blood count and peripheral blood film may reveal mild anaemia, of the normochromic and normocytic type, as is the case in chronic diseases. A markedly raised erythrocyte sedimentation rate (ESR) portends to the possibility of TB. The white cell count is often normal [2].

### 3.2. Radiological Investigations

Ultrasound may reveal discrete of conglomerated (matted) lymphadenopathy. Small discrete anechoic areas, representing zones of caseation, may be seen within the nodes [2]. Calcification in healing lesions is seen as discrete reflective lines [2]. Both caseation and calcification are highly reflective of TB, as opposed to a malignant cause of lymphadenopathy.

Computed Tomography scans may be able to distinguish between extraluminal or intraluminal pathology, as well as disease extent.

### 3.3. Endoscopy/Colonoscopy

Three types of intestinal lesions are commonly seen, ulcerative, stricturous, and hypertrophy. These morphological types may coexist, for example, ulceroconstrictive and ulcerohypertrophic lesions. Crohn's disease typically presents as “skip-lesions,” while both sides of the ileocaecal valve are commonly involved in ileocaecal TB, leading to incompetence of the valve. A finding of ileocaecal valve incompetence may thus help distinguish tuberculosis from Crohn's disease [2].

It is important to distinguish between Crohn's disease and TB, as steroid therapy instituted for Crohn's disease could have disastrous effects on a patient who has EPTB instead.

### 3.4. Ultrasound Guided Fine Needle Aspiration Cytology (FNAC)/Biopsy

FNAC may be useful in guiding to a diagnosis of EPTB. Tissue biopsy specimens are typically more helpful in diagnosing EPTB. Features such as granulomatous inflammation, with epithelioid macrophages, Langhans' giant cells, and lymphocytes, accompanied by characteristic caseation necrosis typify TB infection [2]. However, these features are not pathognomonic for TB and a culture (Ziehl-Neelsen stain for acid fast mycobacterium) is required to establish a laboratory diagnosis of EPTB [2, 10].

### 3.5. Laparoscopy

Laparoscopic findings can be classified into 3 categories: thickened peritoneum with military yellowish white tubercles, only thickened peritoneum, and fibroadhesive pattern. Visual diagnosis and histological diagnosis made on the basis of typical granuloma are comparable, to the experienced eye [2].

### 3.6. Others

Molecular genetics is at the forefront of modern day medicine and a variety of molecular diagnostic tools have been found to be effective in diagnosis EPTB as well. These include Quanti FERON-TB gold (QFT-G), Nucleic Acid Amplification (NAA) assays for Mycobacterium TB DNA, and Polymerase Chain Reaction (PCR) [2, 10].

## 4. Management

### 4.1. Anti-TB Drugs

As with pulmonary TB, the first line of treatment for EPTB is anti-TB medications. Multidrug therapy, including at least 2 of isoniazid, rifampicin, and ethambutol, are considered to be effective in preventing drug-resistant TB [2, 10]. A 6-month course of therapy, including an initial intensive phase, followed by a continuation phase eliminates most of the residual bacilli and reduces the risk of treatment failure and TB recurrence [10]. Directly Observed Treatment, Short Course (DOTS) has also proven to be very effective in ensuring treatment compliance and reducing drug-resistant TB.

### 4.2. Surgery

In most cases of EPTB, medical treatment is curative. Surgery is usually reserved for patients who have developed complications, including perforations, fistulas, massive bleeding, obstruction, or those not responding to medical therapy [2]. The vast majority of patients exhibit rapid symptom improvement, within 2 weeks of anti-TB treatment. Laparotomy may thus be considered, if a patient with EPTB does not appear to respond to treatment beyond this time.

### 4.3. In Pregnancy

A high index of suspicion is required in at-risk populations, such as ethnic minorities, migrant communities, and immunocompromised individuals. Tuberculin skin testing is a valuable screening test in pregnancy and should be carried out if latent TB is suspected. Imaging modalities such as chest X-ray with shielding, CT scan, or ultrasound can be performed as necessary [10].

Pregnancy does not affect the course of TB. However, any delay in diagnosis and treatment could result in catastrophic repercussions to mother and baby, such as the resultant viscus perforations and severe preterm delivery as seen in our case described or even mortality. Intrauterine growth restriction and vertical transmission have also been described [10]. Coinfection with HIV increases maternal mortality rates [10].

First-line antituberculous drugs such as isoniazid, rifampicin, and ethambutol can be used safely in pregnancy and while breastfeeding.

EPTB is an important diagnosis to be considered in high risk women with a nonspecific presentation of abdominal pain and chronic constitutional symptoms, as in our case. Diagnostic delay can lead to unnecessary maternal and fetal morbidity and mortality [11].

In our case described, a pregnant lady presenting with chronic constitutional symptoms should have been thoroughly investigated for possible causes, at primary contact, with referral to the obstetrics and medical teams for specialist care.

Normochromic normocytic anaemia, which does not respond to oral iron therapy, warrants further investigations to look for chronic blood loss, chronic disease, and malignancy. With an earlier referral, a biopsy might have sufficed, with consequent medical anti-TB therapy commenced. Viscus perforation may have been avoided, with resultant less morbidity for both mother and baby.

## 5. Conclusion

Tuberculosis remains an important cause of mortality and severe morbidity in the world, despite the availability of sound Bacillus Calmette-Guerin (BCG) vaccination programmes in most countries. It is often difficult to differentiate EPTB from Crohn's disease or malignancy. In populations where TB is endemic or in marginalized communities such as migrant populations, a high index of suspicion is crucial in diagnosing and appropriately managing EPTB.

Increased vigilance should be afforded to the pregnant population presenting with constitutional symptoms such as drastic weight loss or chronic malaise and night sweats. Early specialist consult should be sought and a thorough investigation made, bearing in mind the possibility of TB in at-risk populations. With early multidisciplinary team involvement and the commencement of appropriate treatment, medical cure rates and prognosis is excellent for patients with EPTB in pregnancy.

## Abbreviations

AFB: Acid fast bacilli
BCG: Bacillus Calmette-Guerin
EPTB: Extrapulmonary tuberculosis
ID: Infectious Disease
TB: Tuberculosis.
